# Comparative Transcriptome Analysis of Leaves and Roots in Response to Sudden Increase in Salinity in *Brassica napus* by RNA-seq

**DOI:** 10.1155/2014/467395

**Published:** 2014-08-07

**Authors:** Hui-Yee Yong, Zhongwei Zou, Eng-Piew Kok, Bih-Hua Kwan, Kingsley Chow, Shiori Nasu, Masami Nanzyo, Hiroyasu Kitashiba, Takeshi Nishio

**Affiliations:** ^1^Graduate School of Agricultural Science, Tohoku University, 1-1 Tsutsumidori Amamiyamachi, Aoba-ku, Sendai, Miyagi 981-8555, Japan; ^2^Molecular Population Genetics Group, Temasek Lifesciences Laboratory, 1 Research Link, National University of Singapore, Singapore 117604; ^3^ACGT Sdn. Bhd. Lot L3-I-1, Enterprise 4, Technology Park Malaysia, 57000 Kuala Lumpur, Malaysia; ^4^National Heart Centre Singapore Pte. Ltd., 17 Third Hospital Avenue No. 01-00, Singapore 168752

## Abstract

Amphidiploid species in the Brassicaceae family, such as *Brassica napus*, are more tolerant to environmental stress than their diploid ancestors.A relatively salt tolerant *B. napus* line, N119, identified in our previous study, was used. N119 maintained lower Na^+^ content, and Na^+^/K^+^ and Na^+^/Ca^2+^ ratios in the leaves than a susceptible line. The transcriptome profiles of both the leaves and the roots 1 h and 12 h after stress were investigated. *De novo* assembly of individual transcriptome followed by sequence clustering yielded 161,537 nonredundant sequences. A total of 14,719 transcripts were differentially expressed in either organs at either time points. GO and KO enrichment analyses indicated that the same 49 GO terms and seven KO terms were, respectively, overrepresented in upregulated transcripts in both organs at 1 h after stress. Certain overrepresented GO term of genes upregulated at 1 h after stress in the leaves became overrepresented in genes downregulated at 12 h. A total of 582 transcription factors and 438 transporter genes were differentially regulated in both organs in response to salt shock. The transcriptome depicting gene network in the leaves and the roots regulated by salt shock provides valuable information on salt resistance genes for future application to crop improvement.

## 1. Introduction

Soil salinity is widespread throughout the globe, with more than 20 million hectares of world's land being estimated to be salt-affected [1–3]. It has been reported that 45 million ha of the current 230 million ha of irrigated land and 32 million ha of the 1,500 million ha under dryland agriculture are salt-affected [3]. Salinity occurs through natural or human-induced processes that result in the accumulation of dissolved salts. Particularly, salinity resulting from natural disasters such as tsunamis often has an immediate impact on agricultural land due to a sudden substantial increase in soil salinity level. The Great East Japan Earthquake, which occurred in 2011, inundated more than 20,000 ha of agricultural land, resulting in substantial reduction of crop production [4].

Salt tolerance is a very complex phenomenon in most plant species since it involves various mechanisms at cellular, tissue, organ, or whole plant levels. Stress exposure at different development stages affects different pathways for adaptation and homeostasis resulting in different gene expression profiles. Over the past several decades, germplasms of the Brassicaceae oilseed crops have been screened for salt tolerance, and some elite lines have been identified [5–7]. Based on various studies of salt tolerance in* Brassica* species, the amphidiploid species in the triangle of U [8], that is,* Brassica carinata*,* Brassica juncea,* and* Brassica napus*, outshone the diploid species, that is,* Brassica rapa*,* Brassica nigra*, and* Brassica oleracea* [9–11]. Comparative salt tolerance study of* Brassica* species has also revealed that* B. napus* is more salt tolerant than the other amphidiploids, that is,* B. carinata* and* B. juncea* [12].

Gene expression response varies with time from stress exposure. Recent transcriptomic research on plant salt tolerance has been gradually shifting from salt-sensitive glycophytes to salt tolerant halophytes. The halophyte species which has been extensively surveyed for the transcriptomic response to salt is salt cress (*Thellungiella halophila*) [13, 14]. Recently, the salt-responsive transcriptome of a semimangrove plant (*Millettia pinnata)*, which is a glycophyte with moderate salt tolerance, has also been thoroughly characterized and study has revealed various affected pathways [15]. Although transcriptome analysis in salinity response has been extensively conducted in these two species, the salt-responsive transcriptomic regulation of salt tolerant polyploid species is still worth unveiling since their transcriptome architecture is more complex than that of diploid species and they can generally withstand adverse environmental conditions better than their diploid ancestors.

The advent of next-generation sequencing (NGS) technologies has progressively revolutionized genomic studies. RNA-seq technologies have been employed for studying both model and nonmodel organisms. For model organisms, in which both genome sequences and gene annotations are available, a protocol for differential gene and transcript expression analysis of RNA-seq experiments described by Trapnell et al. is suitable for conducting the transcriptome analysis [16]. For nonmodel organisms, deep sequencing followed by* de novo* assembly and clustering is necessary to generate reference transcriptome. Alternatively, genome sequences and expressed sequence tag (EST) sequences of related species can be utilized as references for mapping. Hybridization-based microarray technologies have been the dominant approaches for the study of gene expression in the past decade. However, these methods suffer from several limitations including reliance upon existing information about the available genome sequence, high background levels attributed to cross-hybridization, and a limited dynamic range of detection owing to both background and saturation of signals [17, 18]. Conversely, RNA-seq offers several advantages and is much superior to the microarray technologies because of a wider range of expression levels, less noise, higher throughput, more information to detect allele-specific expression, novel promoters, splice variants and isoforms, and no necessity of prior knowledge of both genome and gene sequences [19, 20].

Numerous studies have been performed to discover genes that contribute to salinity tolerance in* B. napus* [21–25]. However, only a limited number of genes have been evaluated and these are not sufficient to characterize the overrepresented molecular mechanism underlying moderate salt tolerance. In this study, we commenced a comprehensive transcriptome analysis of a* B. napus* line, which is one of the most salt tolerant lines obtained in our prior screening [7]. In Japan, a sudden increase in soil salinity due to tsunami has been the major problem in agriculture sector [4]. To grow oilseed rape or other brassica species, farmers would either directly sow seeds into soil or transplant germinated seedlings into soil. Plants experience sudden increase in salinity after transplantation into salt contaminated land and this condition is more akin to salt shock that has been described by Shavrukov [26]. Taji et al. also suggested that rapidly inducible genes should be important for salt tolerance [14]. Therefore, this study focused on the initial transcriptome regulation in leaves and roots of this line in response to sudden increase in salinity. Transcriptomic changes in this line were evaluated by comparing the leaf and root expression profiles at 1 h and 12 h time points of salt challenge.

## 2. Materials and Methods

### 2.1. Plant Materials

Seeds of the* B. napus* line N119 maintained in the Tohoku University* Brassica* Seed Bank were surface-sterilized with 70% ethanol and 1% sodium hydrochlorite and germinated on MS medium. Germinated seedlings were grown in plastic pots (diameter = 10.5 cm, height = 9 cm) containing vermiculite under a 16/8 h photoperiods at 23°C. Plants were watered twice a week with 1/2000 HYPONeX fertilizer solution (Hyponex, Osaka, Japan) at a final concentration of 30 *μ*g/L nitrogen, 20 *μ*g/L phosphorus, and 25 *μ*g/L potassium. Salt treatment was implemented when the seedlings were 3 weeks old. The volume of each plant pot was approximately 800 mL. For salt treatment, plants were watered by 200 mM of NaCl solution. The treatments were initiated at 8.00 a.m. and each individual plant was watered with 300 mL of salt water to field capacity. Meanwhile, a group of control plants were watered with an equal volume of distilled water.

### 2.2. Analysis of Ion Contents

For the quantitative analysis of ions, that is, Na^+^, K^+^, and Ca^2+^, the aerial parts of the seedlings were dried at 85°C for 10 days. Dried samples were ground into powder using tissue lyzer, MiXer MiLL MM300 (QIAGEN Inc., USA), and 20 mg of the powder was digested with 5 mL of 1 M hydrochloric acid overnight and filtered using Whatman 42 mm paper. Ion contents were estimated using an A-2000 atomic absorption spectrophotometer (Hitachi High-Technologies, Japan). Standard solutions of Na^+^, K^+^, and Ca^2+^ were used for calibration.

### 2.3. Sample Collection, RNA Preparation, and Sequencing

The roots and leaves were sampled at 1, 3, 6, 12, and 24 h after stress with three replicates for each time point. Both the whole root and the third leaf were sampled simultaneously from each individual plant and were separately frozen in liquid nitrogen and stored at −80°C prior to RNA isolation. The total RNA was extracted using SV Total RNA Isolation System (Promega, Madison, WI, USA) following the manufacturer's instructions. The quality of the RNA was determined by NanoDrop 1000 spectrophotometer (Thermo Scientific, Wilmington, USA) and a 2100 Bioanalyzer RNA Nanochip (Agilent Technologies GmbH, Berlin, Germany). The expression of salt-responsive genes was analyzed for each sample in order to determine the suitable time point for samples to be sent for sequencing (Additional file 1 in Supplementary Material available online at http://dx.doi.org/10.1155/2014/467395). The total RNA samples of three replicates for each organ and each time point were bulked. At least 20 *μ*g of total RNA samples of both the root and leaf tissues (both salt treated and control) collected at 1 h and 12 h after stress was sent to the Beijing Genomics Institute, Hong Kong, for commercial Illumina sequencing.

mRNAs were purified using oligo(dT)-attached beads and fragmented into small pieces (100–400 bp). The cleaved RNA fragments were then primed with random hexamers and subjected to the synthesis of the first-strand and second-strand cDNAs. The synthesized cDNAs were ligated with paired-end adaptors. The cDNA fragments with 200 bp (+20 bp) size were then selected by agarose gel electrophoresis and enriched by PCR amplification. Finally, eight cDNA libraries were constructed for sequencing on an Illumina HiSeq2000 sequencer. Reads for all eight transcriptomes of* B. napus* are available through the NCBI Sequence Read Archive (SRA), study accessions (GenBank: SRP028575).

### 2.4. *De Novo* Assembly and Sequence Clustering

The raw reads were cleaned by trimming adapter sequences, low-quality sequences (reads with ambiguous bases “N”), and reads with more than 10% *Q* < 20 bases.* De novo* assembly of the clean reads was performed using Trinity [27] with default setting with an optimized k-mer length of 25 and the scaffolds obtained were denoted as unigenes. The Trinity unigenes of eight libraries were then further clustered into a comprehensive transcriptome using CD-HIT-EST software with a sequence identity cut-off of 0.9 and comparison of both strands [28]. For comparison, SOAPdenovo (version 1.04; http://soap.genomics.org.cn/soapdenovo.html) [29] was also used to conduct* de novo* assembly with default setting except for the k-mer value, which was set at specific values of 29, 31, 33, 35, 37, 39, 41, 43, 45, 47, 49, 51, 53, 55, 57, 59, 61, and 63. SOAPdenovo assembly with k-mer of 51 produced the highest N50 value and average scaffold length. Therefore, SOAPdenovo unigenes produced by k-mer of 51 were used for further clustering analysis by CD-HIT-EST.

Both all-unigenes of Trinity and SOAPdenovo were searched against NCBI* Brassica napus* nonredundant unigene sequences with an* E* value cut-off of 1.0 × 10^−5^. The Trinity all-unigenes corresponded to 61,015* Brassica napus* unigene sequences, while the SOAPdenovo all-unigenes got fewer hits, that is, 58,357 sequences. Therefore, Trinity all-unigenes were used for subsequent analysis.

### 2.5. Expression Analysis and Identification of DEGs

Clean reads of eight transcriptomes were mapped back to all-unigenes with RSEM v1.2.3 [30] allowing the maximum 3 mismatches. The reads per kilobase of exon per million mapped reads (RPKM) values were applied to measure the gene expression levels. For a given all-unigene, eight RPKM values were generated from eight transcriptomes, respectively. DEGs between control and salt-water treated samples were identified by EBSeq R package v1.1.5 [31]. Since biological replicates were pooled, transcript specific variance was determined by estimating the across-condition variance as recommended in the vignette [32]. Salt-responsive genes were identified with a FDR<0.01 and a normalized fold change ≥2.

### 2.6. Functional Categorization and Annotation

To assign gene ontology annotation for all-unigenes, the all-unigenes were aligned to SwissProt database using BLASTX with an *E* value cut-off of 1.0 × 10^−5^. The results with the best hits were extracted. The all-unigenes without SwissProt hits were searched against the NCBI NR protein database by BLASTX with an *E* value cut-off of 1.0 × 10^−5^. The GO annotations for the top blast hits were retrieved with the Blast2GO program [33, 34], followed by functional classification using WEGO software [35]. In addition, KEGG ontology was assigned to each of the all-unigenes by KOBAS 2.0 (KEGG Orthology-Based Annotation System, v2.0), in which all-unigenes were aligned to the KO database by BLASTX with an *E* value cut-off of 1.0 × 10^−5^ to retrieve gene IDs, followed by ID mapping to KO terms [36]. Furthermore, all-unigenes were searched against Plant Transcription Factor Database v2.0 (PlantTFDB 2.0) [37] and the Transporter Classification Database (TCDB) [38, 39] with an *E* value cut-off of 1.0 × 10^−5^ and more than 80% query coverage.

The GO enrichment analysis and KEGG pathway enrichment analysis were analyzed by BiNGO plugins for Cytoscape, using the hypergeometric test for statistical analysis with the whole* B. napus* transcriptome as the background [40]. For *P* value correction, the rigorous Bonferroni correction method was employed. The cut-off *P* value after correction was 0.05.

### 2.7. RT-PCR and Real-Time Quantitative PCR

The gene-specific primers for real-time PCR analysis were designed using Primer 3 by applying the parameters described by Thornton and Basu [41]. We conducted RT-PCR for 40 DEGs using* B. napus* actin gene as control (Additional file 1). The first-strand cDNAs were synthesised from 1 *μ*g of total RNAs using SuperScript III Reverse Transcriptase (Invitrogen, Carlsbad, CA). Ten microliters of PCR samples containing 1 *μ*L of first-strand cDNAs and 5 pmol of primers were then subjected to 30 cycles of 30 s denaturing at 94°C, 30 s annealing at 60°C, and 30 s extending at 72°C. The PCR products were electrophoresed on 1.5% agarose gel.

Real-time PCR was performed on CFX Connect Real-Time PCR Detection System (Bio-Rad, Hercules, CA) using 1 *μ*L of cDNAs and SsoAdvanced SYBR Green Supermix (Bio-Rad). The thermal conditions were set at 95°C for 3 min denaturation, followed by 40 cycles of 95°C for 1 s and 60°C for 30 s. Following denaturation at 95°C for 30 s and cooling to 65°C for 30 s, a melting curve was generated by heating from 65°C to 95°C in 0.5°C increments with a dwell time at each temperature of 2 s while continuously monitoring the fluorescence. All of the reactions were performed in triplicate and the average expression value was calculated. The relative expression level for each gene was calculated using the 2^−ΔΔCT^ method with normalization to the internal control gene [42].

## 3. Results and Discussion

### 3.1. Ion Contents in a Salt Tolerant Line and a Salt Susceptible Line

**Figure 1:**
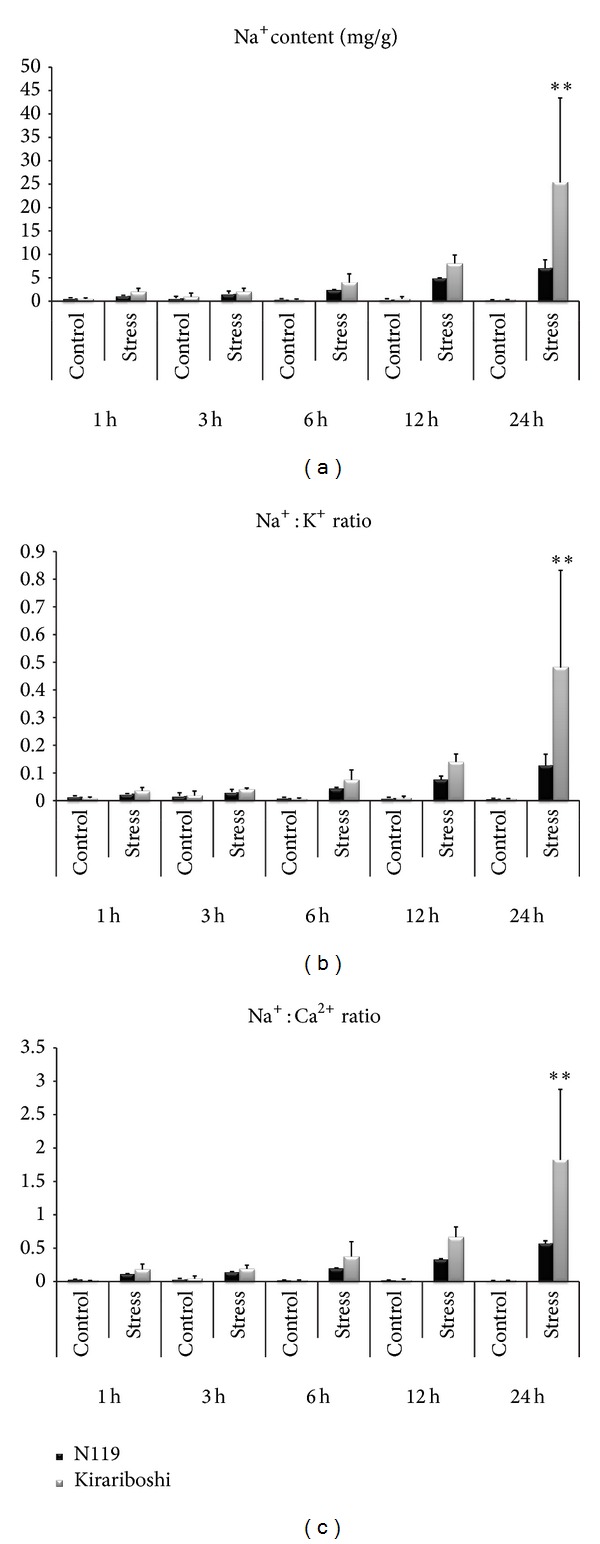
Ion contents in the leaves of* B. napus* in response to salt stress. Symbol “∗∗” indicates significant difference at *P* < 0.01 by *t*-test.

Transcriptome of a salt tolerant line, N119 of cv. “Sapporo,” which has been evaluated as being one of the most salt tolerant lines in our previous study [7], was investigated. Based on our previous study [7], salt tolerance for various* B. napus* lines has been screened by irrigating 3-week-old seedlings with 200 mM NaCl and the degree of salt tolerance has been determined by the ratio of dry weights of plants grown with NaCl to those of plants grown without NaCl. N119 has been identified as tolerant line due to its high dry weight ratio [7]. To further confirm the salt tolerance of N119, seedlings of N119 together with a susceptible line, “Kirariboshi,” for comparison were subject to short-term salt stress, and their responses were analyzed by measuring Na^+^, K^+^, and Ca^2+^ contents. Instead of alleviation of biomass reduction, the maintenance of a low Na^+^ content and a low Na^+^/K^+^ ratio has been widely used as an index of salt tolerance [43]. An increase of the period of exposure to salinity resulted in an increase in the Na^+^ content, the ratio of Na^+^/K^+^, and the ratio of Na^+^/Ca^2+^ in both lines (Figure 1). However, N119 seedlings performed different from those of “Kirariboshi.” The Na^+^/K^+^ ratio and Na^+^/Ca^2+^ ratio of “Kirariboshi” reached ~2-fold that of N119 12 h after salt treatment and significant differences were observed at 24 h after stress (Figures 1(b) and 1(c)). The lower Na^+^/K^+^ ratio maintained by N119 should have conferred a less stressed cellular environment than that by “Kirariboshi.” This response agreed with the previously published observations, in which both salt tolerant rice and foxtail millet cultivars were found to maintain a low Na^+^/K^+^ ratio in their shoot tissues [43, 44].

### 3.2. Time Point for Transcriptome Analysis

The expression levels of four salt-responsive genes, that is,* BnBDC1* [22],* BnLEA4* [23],* BnMPK3* [45], and* BnNAC2* [46], were analyzed in order to determine the most appropriate time point(s) for transcriptome analysis of N119. Generally, the expression showed more than 2-fold upregulation at 1 h after stress and subsequent downregulation at the following time points (Additional file 2). However, some genes were observed to maintain the upregulated expression at 12 h and 24 h after stress. Together with the phenotypic data, that is, Na^+^ content, the ratio of Na^+^/K^+^, and the ratio of Na^+^/Ca^2+^, transcriptome regulation of salt stress was analyzed at 1 h and 12 h after stress.

### 3.3. Nucleotide Sequencing and* De Novo* Assembly

Although nonredundant unigenes of* B. napus* are available at the National Center for Biotechnology Information (NCBI), the coverage of these data in the whole transcriptome of* B. napus* is uncertain. Since* B. napus* whole genome sequences and annotations of* B. napus* are not available at present, reference-based transcriptome analysis is also not feasible for* B. napus*. Therefore,* de novo* assembly appears to be a good approach to study salt regulated expression changes in this species.

For a broad survey of salt-responsive genes, eight cDNA libraries were prepared from mRNA from the leaves and the roots of the control and salt-treated plants, denoted as* BnLc* (leaves of control plants),* BnLs* (leaves of salt-treated plants),* BnRc* (roots of control plants), and* BnRs* (roots of salt-treated plants) sampled at 1 h and 12 h and sequenced by Illumina deep-sequencing. After removal of low quality and adapter sequences, nearly 211 million clean reads remained for all eight transcriptomes. The percentages of Q20 bases for the clean reads in all eight transcriptomes were all above 96% (Additional file 3). In sum, the clean reads constituted ~38 GB of sequence data.

**Figure 2:**
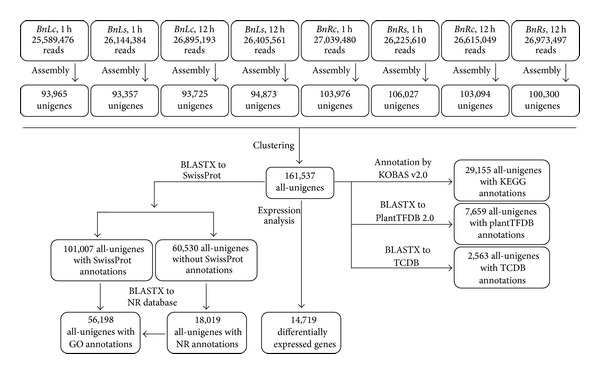
Flowcharts of the transcriptome analysis for* B. napus.* The whole analysis involved sequencing assembly by Trinity and clustering by CD-HIT-EST, SwissProt/Nr annotation, GO annotation, KEGG annotation, aligning to PlantTFDB 2.0 and TCDB, and identification of DEGs as candidate salt-responsive genes.

*De novo* assembly was carried out by the Trinity method [27] and nonredundant sequences were obtained by clustering using CD-HIT-EST [28] (Figure 2). From now on, the clustered unigene sequences are herein referred to as all-unigenes. Overviews of assembly results are shown in Tables 1 and 2. These sequence reads were finally clustered to 161,537 nonredundant all-unigenes, spanning a total of 112 Mb of sequences (Table 3, Figure 2). All the all-unigenes were longer than 200 bp. Mean length and N50 of the final all-unigenes were 693 bp and 1,039 bp, respectively. By the Trinity* de novo* assembly method, no “N,” that is, unidentified nucleotide, remained in the final unigenes. Due to an unexpectedly large number of all-unigenes obtained after clustering,* de novo* assembly was performed again by the SOAPdenovo program version 1.04 [29]. Clustering of the SOAPdenovo unigene sequences yielded 191,237 nonredundant sequences. Nevertheless, the assembly quality was worse than that by the Trinity method. All-unigenes generated by SOAPdenovo had a mean length of 506 bp and N50 of 592 bp, and 25% of the all-unigenes had at least one “N” (Additional file 4). The results were similar to those of transcriptome assembly reports of* Aegilops variabilis* [47] and* Chorispora bungeana* [48], in which the assembly qualities of the Trinity method were superior to those of the SOAPdenovo method. All-unigenes generated by both Trinity and SOAPdenovo were searched against the* B. napus* nonredundant unigenes from NCBI. The Trinity all-unigenes corresponded to 61,015* B. napus* nonredundant unigenes from NCBI, while the SOAPdenovo all-unigenes had 58,357 hits. Therefore, the assembly results from the Trinity method were used for all of the following analyses.

### 3.4. Functional Annotation of All-Unigenes of* Brassica napus*

Annotation of all-unigenes was performed by searching them against the SwissProt database. Among the 161,537 all-unigenes, 101,007 (62.5%) had at least one hit to the SwissProt database in BLASTX search with *E* value ≤1 × 10^−5^ (Additional file 5, sheet 1). The NCBI nonredundant (NR) protein database was searched for the remaining all-unigenes without a SwissProt hit and 18,019 (11.2%) of all-unigenes showed significant similarity to their respective subjects at *E* value ≤1 × 10^−5^ (Additional file 5, sheet 1). Overall, these all-unigenes matched 41,169 unique protein accessions (30,401 for SwissProt and 10,768 for NR hit). Only 57.9% of the all-unigenes shorter than 500 bp had BLAST hits in either the SwissProt or NR database (Additional file 6). The proportion of all-unigenes hit with BLAST increased markedly in those with larger sizes.

Functional classifications of gene ontology (GO) terms of all unigenes are shown in Additional file 6. In total, 56,198 out of 119,026 all-unigenes with either SwissProt hits or NCBI NR hits were assigned to GO terms (Figure 2). In the category of “Biological Process,” the largest groups were of “cellular process,” “metabolic process,” “biological regulation,” and “response to stimulus” (Additional file 7). In the category of “Molecular Function,” “binding” and “catalytic” activities were the largest group. In the category of “Cellular Component,” most of the all-unigenes were located in “cell” and “organelle.” In order to further understand the biological function and interaction of genes, pathway-based analysis was performed based on the Kyoto Encyclopedia of Genes and Genome (KEGG) Pathways database, which documents the networks of molecular interaction in the cells and variants of them specific to particular organisms. All-unigenes were mapped against the KEGG Ontology (KO) database by BLASTX. Mapped all-unigenes were annotated by KOBAS v2.0 [36]. We performed KEGG pathway analysis to assign the all-unigenes to biological pathways. In total, 29,155 all-unigenes were assigned to 245 pathways. These pathways belonged to 25 clades under 5 major KEGG categories, that is, “Metabolism,” “Genetic information processing,” “Cellular process,” “Environmental information processing,” and “Organism systems” (Additional file 8). Among these pathways, “plant hormone signal transduction,” “spliceosome,” “oxidative phosphorylation,” “RNA transport,” and “protein processing in endoplasmic reticulum” were the top five pathways represented by all-unigenes.

**Figure 3:**
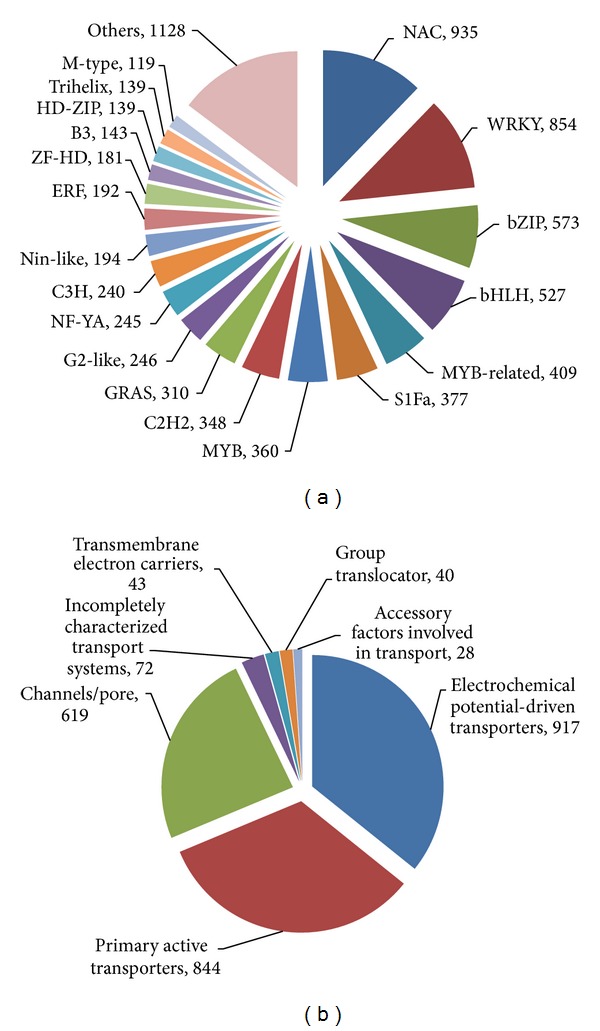
Classification of transcription factor and transporter gene families in* B. napus.* (a) Distribution of transcription factor gene families in* B. napus*. (b) Distribution of transporter gene families in* B. napus*.

Searching against the Plant Transcription Factor Database v2.0 (PlantTFDB 2.0) [37] matched 7,659 all-unigenes to 57 unique transcription factor (TF) gene families (Figure 3(a); >Additional file 4, sheet 2). In total, these putative* B. napus* transcription factor genes represent 4.72% of the total transcripts. The overall percentage distribution of transcripts encoding transcription factors among the various known protein families is similar to those in* Arabidopsis thaliana* and* B. rapa* published earlier [37] (Additional file 9). However, the number of genes increased for a few families, such as NAC, WRKY, S1Fa-like, GRAS, NF-YA, Nin-like, and ZF-HD. Interestingly, some transcription factor families absent in* B. rapa* were found in* Brassica napus* in this study, for example, NF-X1, CAMTA, CPP, HB-PHD, and SAP. These observations indicate the evolutionary significance among these species. In addition, a BLASTX search against the transporter classification database (TCDB) [38, 39] identified 2,563 transporter genes in all-unigenes (Figure 3(b); Additional file 5, sheet 3). The majority of the transporter genes belonged to “Electrochemical potential-driven transporters,” “Primary active transporters,” and “Channel/pores.”

### 3.5. Identification of DEGs

Two criteria of screening threshold were applied to identify differentially expressed genes (DEGs); that is, (i) the average of fold change in gene expression level was more than or equal to 2-fold between salt-treated and distilled water-treated samples and (ii) the false discovery rate (FDR) was less than 0.01. Under these criteria, 14,719 out of 161,537 all-unigenes were found to be differentially expressed in at least one tissue at one condition (Additional file 10). Overall, the number of DEGs was greater in the roots (8,665 DEGs) than those in the leaves (7,795 DEGs) (Additional file 11). There were also many more DEGs which responded at 1 h after stress (9,242 DEGs) than those at 12 h (7,635 DEGs). In both the leaves and the roots, the number of upregulated DEGs by salt treatment was more prominent than that of downregulated DEGs. Only a subset of DEGs shared a common tendency of expression changes between both organs, that is, 432 upregulated and 110 downregulated at 1 h after stress and 133 upregulated and 134 downregulated at 12 h after stress (Additional file 11). A relatively small portion of DEGs showed an opposite tendency of expression changes between the two organs, that is, 120 DEGs upregulated in the leaves but downregulated in the roots and 96 DEGs downregulated in the leaves but upregulated in the roots at 1 h after stress; 115 DEGs upregulated in the leaves but downregulated in the roots and 97 DEGs downregulated in the leaves but upregulated in the roots at 12 h after stress. The remaining majority of DEGs were distinctly upregulated or downregulated in either the leaves or the roots.

### 3.6. Validation of DEGs by Semi qRT-PCR and Real-Time qPCR

**Figure 4:**
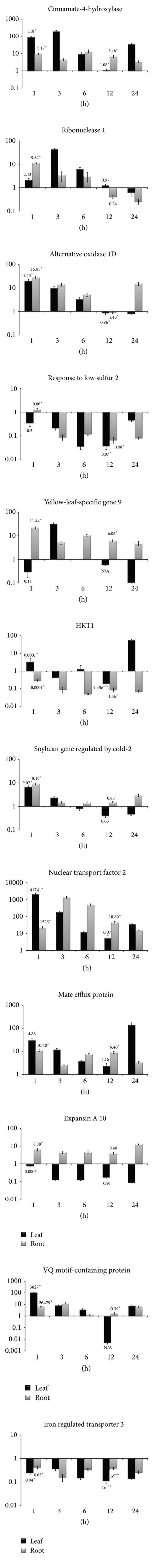
Validation of DEGs with qRT-PCR. The *x*-axis represents hours after stress while the *y*-axis represents salt-induced expression fold change relative to control treatment (0 mM). The number of label above the bar is fold change obtained from RNA-seq data. Symbol “∗” indicates FDR < 0.01.

To validate the reliability of our sequencing approach in identifying salt-responsive DEGs, 40 randomly selected DEGs for eight conditions, that is, 23 DEGs at 1 h and 13 DEGs at 12 h in the leaves and 26 DEGs at 1 h and 13 DEGs at 12 h in the roots, were tested by semi qRT-PCR (Additional file 12). These DEGs consisted of previously discovered salt-responsive genes, genes encoding ion transport proteins, and novel salt-responsive genes. The novel salt-responsive genes tested in this study were* Nuclear Transport Factor 2*,* PQ-loop Repeat Family Protein*,* Response to Low Sulfur 2*,* Yellow-Leaf-Specific Gene 9*,* Ribonuclease 1*,* VQ-motif Containing Protein*, and some unknown proteins. The semi qRT-PCR profiles of these DEGs were basically in agreement with the RNA-seq results. Among these DEGs, expression trends of 12 DEGs were further evaluated by real-time qRT-PCR at 1 h, 3 h, 6 h, 12 h, and 24 h (Figure 4). Although the expression fold change differed a little between the RNA-seq and qRT-PCR, the patterns were similar.

To investigate whether Kirariboshi has the similar expression fold change of salt-responsive DEGs to that of N119, qRT-PCR analyses for some DEGs were carried out. The result indicated that Kirariboshi showed similar regulation of the expression fold change for most of the DEGs to that in N119 (Additional file 13).

### 3.7. Functional Characterization of DEGs

To further characterize the expression changes in these two organs at two time points, GO enrichment analysis was conducted for the DEGs with the whole transcriptome set as background. The enriched GO terms were also compared between upregulated and downregulated DEGs at each time point after the salt treatment of both organs (Additional files 14–17). In the roots, as the first organ exposed to salt stress, at 1 h after stress, the top five overrepresented GO terms of “Biological Process” for upregulated DEGs were “response to water deprivation,” “response to abscisic acid stimulus,” “response to chemical stimulus,” “hyperosmotic salinity response,” and “response to organic substance” (Additional file 14, sheet 5). Overrepresented GO terms existing in upregulated DEGs at both 1 h and 12 h after stress in the roots were “response to wounding,” “response to chemical stimulus,” “response to organic substance,” “regulation of response to stimulus,” “response to stimulus,” “response to chitin,” “response to stress,” and “oxidoreductase activity” (Additional files 14–17). At 1 h after stress, in the leaves, the top five significantly overrepresented GOs for upregulated DEGs were “response to chitin,” “response to abscisic acid stimulus,” “response to organic substance,” “hyperosmotic salinity response,” and “response to jasmonic acid stimulus” (Additional file 14, sheet 1). Some overrepresented GOs at 1 h after stress in the leaves remained overrepresented for that upregulated at 12 h after stress, for example, “response to abiotic stimulus,” “response to osmotic stress,” “response to cold,” “hyperosmotic salinity response,” “disaccharide transport,” “oligosaccharide transport,” and “response to abscisic acid stimulus” (Additional file 14). However, there were also a number of overrepresented GOs upregulated at 1 h after stress in the leaves that became overrepresented in all-unigenes downregulated at 12 h after stress, for example, “respiratory burst,” “response to chitin,” “response to mechanical stimulus,” “intracellular signal transduction,” “cellular ketone metabolic process,” “defense response,” and “organic acid metabolic process” (Additional file 14). When enriched GO terms of each condition were compared with each other, upregulated DEGs at 1 h after stress in both the leaves and the roots shared the greatest number of the same overrepresented GO terms (49 GO terms) (Additional files 14–17). This indicates that genes of similar functions probably affecting similar pathways were simultaneously regulated and, in this case, upregulated in both the leaves and the roots to overcome salt stress.

Little overlap was observed between the DEGs in the leaves and the roots at both time points. Similar observation has been found in the salt-responsive transcriptome in* M. pinnata* [15]. Although similar pathways could be affected by salt shock in both organs, certain salt-induced detrimental impacts varied between different parts of a plant. The GO enrichment analysis indicated that many distinct groups of genes were activated exclusively in either the leaves or the roots possibly to overcome salt-inducible damages. For example, at 1 h after stress, many phytohormone related pathways were enriched solely in the leaves, that is, “ethylene mediated signaling pathway,” “regulation of gibberellins biosynthetic process,” and “salicylic acid biosynthetic process” (Additional files 14–17). Conversely, in the root at 1 h after stress, genes involved in synthesis of the cellular component and defense response were distinctly overrepresented, that is, “cell wall assembly,” “cell wall macromolecule catabolic process,” “regulation of defense response,” and “regulation of immune system process” (Additional files 14–17).

**Figure 5:**
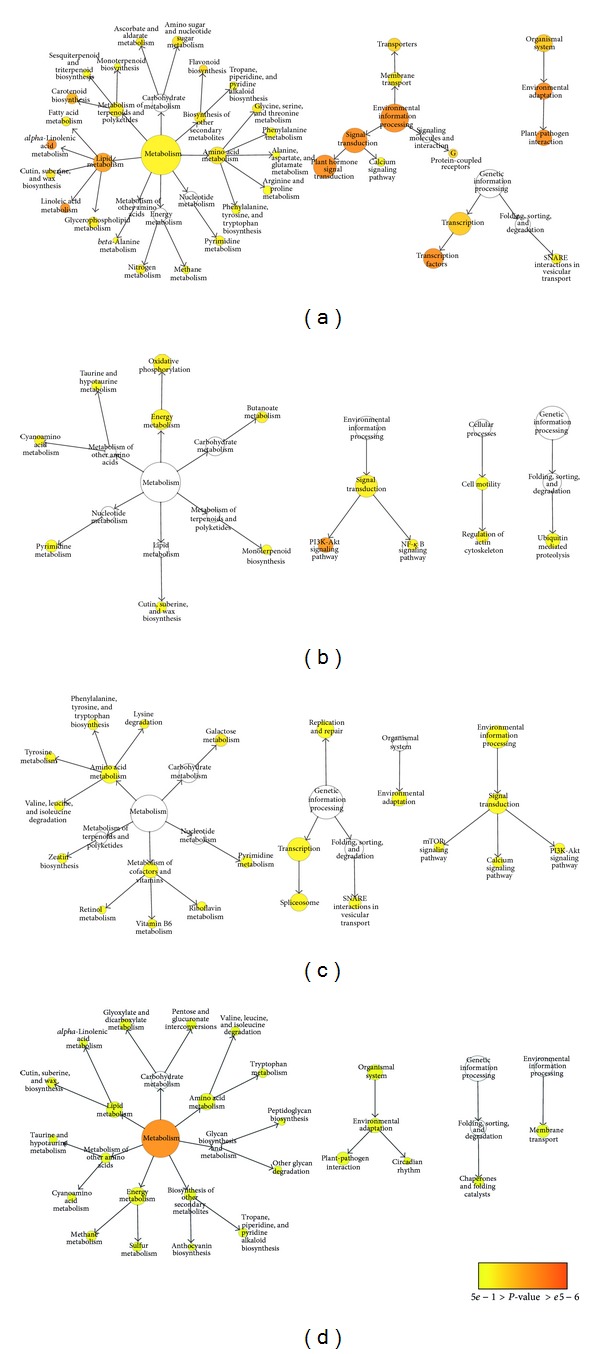
Overrepresented KEGG pathways for DEGs in the leaves. Overrepresented pathways were identified in upregulated DEGs at 1 h after stress (a), downregulated DEGs at 1 h after stress (b), upregulated DEGs at 12 h after stress (c), and downregulated DEGs at 12 h after stress (d), respectively.

**Figure 6:**
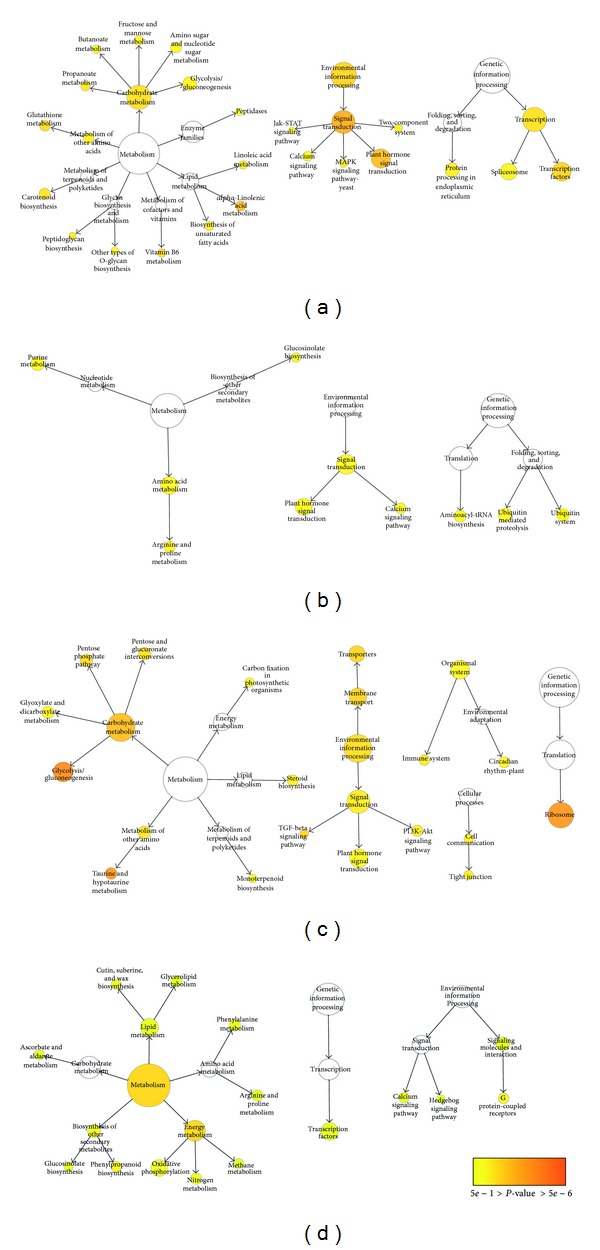
Overrepresented KEGG pathways for DEGs in the roots. Overrepresented pathways were identified in upregulated DEGs at 1 h after stress (a), downregulated DEGs at 1 h after stress (b), upregulated DEGs at 12 h after stress (c), and downregulated DEGs at 12 h after stress (d), respectively.

KEGG pathway enrichment analysis was also performed to further understand the biological meanings of the response time points of transcripts. Enrichment was considered to be significant at corrected *P* value < 0.05. In total for both time points, the DEGs were enriched in 19 ontologies and 23 ontologies in the leaves and in the roots, respectively, at corrected *P* value < 0.05 (Additional file 18; Figures 5 and 6). The KEGG category of “Environmental information processing” was significantly enriched in upregulated root DEGs at both 1 h and 12 h after stress (Additional file 18, sheets 5 and 7; Figures 6(a) and 6(c)). However, different pathways in this category were enriched in the respective upregulated root DEGs at both time points, that is, “plant hormone signal transduction” at 1 h after stress and “transporters” and “TGF-beta signaling pathway” at 12 h after stress. Besides, many pathways in the category of “Metabolism” were enriched at both time points in the roots. In the category of “Metabolism,” the clade of “Carbohydrate metabolism” was overrepresented in the roots at both time points (Additional file 18, sheets 5 and 7). The overrepresentation of “Carbohydrate metabolism” was due to upregulation of several genes involved in “glycolysis/gluconeogenesis,” “fructose and mannose metabolism,” “butanoate metabolism,” “amino sugar and nucleotide sugar metabolism,” and “propanoate metabolism” at 1 h after stress and enrichment of “glycolysis/gluconeogenesis,” “pentose phosphate pathway,” and “pentose and glucuronate interconversions” at 12 h after stress. The changes in KO enrichment in the roots at 1 h and 12 h after stress depicted switches in functional pathway regulation at different time points for salinity adaptation in the roots. In the leaves, the number of overrepresented KO terms was more prominent at 1 h after stress than that at 12 h (Additional file 18, sheets 1–4; Figure 5). At 1 h after stress, a total of seven KO terms significantly enriched in the leaves were also found to be overrepresented in the roots at 1 h after stress (Additional file 18, sheets 1 and 5; Figures 5(a) and 6(a)). This result directly agreed with the GO enrichment analysis, in which both the leaves and the roots shared the most enriched GO terms at 1 h after stress. Generally, the overrepresented KO terms shared between the leaves and the roots at 1 h belong to the clades of “Lipid metabolism,” “Metabolism of terpenoids and polyketides,” “Signal transduction,” and “Transcription.” The KEGG enrichment analysis depicted common, tissue-specific, and time-point-specific patterns of overrepresentation. Overall, this observation demonstrated that various biological substances and signaling molecules are required to cope with salt stress. As more genes were differentially expressed in both organs and more overrepresented GO and KO terms were shared between both organs at 1 h after stress than that at 12 h, DEGs regulated at 1 h after stress were particularly important for salinity adaptation and probably for salt tolerance as well.

### 3.8. Transcription Factors

**Figure 7:**
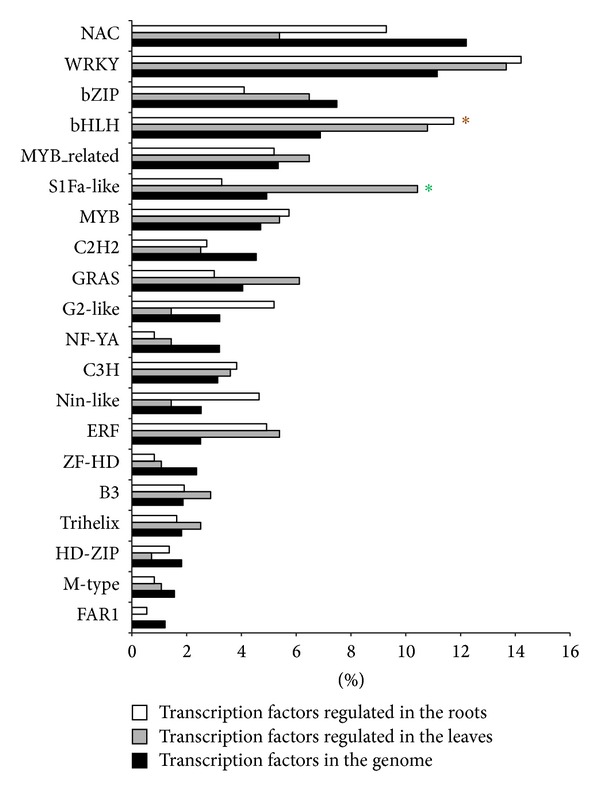
Transcription factors families differently expressed in response to salt stress. The percentage means the fraction of the particular transcription factor in the total transcription factors identified in the whole transcriptome or the fraction of certain transcription factors in those regulated in the roots or the leaves. Symbol “∗” indicates overrepresented transcription factor by hypergeometric test at corrected *P* value < 0.05.

Various transcription factors (TFs) such as AREB/ABF, MYB, AP2/EREBP, bZIP, MYC, HSF, DREB1/CBF, NAC, HB, and WRKY have been shown to orchestrate stress responsive pathways in plants [49, 50]. Based on the putative annotation assigned by homology search with genes in PlantTFDB 2.0, 582 transcription factors representing 45 different families were found to be differentially regulated in both the leaves and the roots at the early stage of salt stress, but only the top 20 differentially regulated TF families are herein shown (Figure 7). These TF families were compared between the leaves and the roots and enrichment analysis was performed to identify families playing vital roles in early stress response.

In the leaves, most of the regulated transcription factors belonged to WRKY, bHLH, and S1Fa-like, in which the S1Fa-like transcription factor family was overrepresented (Figure 7). S1Fa binds to a cis-element within both the cauliflower mosaic virus 35S promoter and the promoter of* rps1*, encoding plastid ribosomal protein S1, and negatively regulates their activity [51]. Other than these two promoters, there was no finding of a novel target regulated by this class of transcription factor. A relatively large number of S1Fa-like transcription factors, that is, 22 genes, were found to be upregulated at 1 h after stress in the leaves of* B. napus*. It would be interesting to elucidate the salt-responsive target genes potentially regulated by this transcription factor family.

In the roots, the majority of the regulated transcription factors belonged to WRKY, bHLH, and NAC, in which bHLH was overrepresented among the DEGs (Figure 7). These upregulated DEGs annotated as bHLH showed high homology to* Arabidopsis ICE2*,* ROX1,* and some bHLH genes from* B. rapa*. In* Arabidopsis*, the expression of a bHLH transcription factor, identified as inducer of CBF expression 1 (*ICE1*), was upregulated by salt stress [52]. Two* Arabidopsis* bHLH transcription factors, that is,* ICE1* and* ICE2*, were discovered to regulate the transcription of* CBF3* and* CBF1*, respectively, under cold stress. Overexpression of* ICE1* and* ICE2* enhances the expression of* CBF3* and* CBF1* and, in turn, improves freezing tolerance [52, 53]. Besides, two homologues of* ICE*, that is,* OrbHLH001* and* OrbHLH2,* from wild rice (*Oryza rufipogon*) are salt-inducible and overexpression of these two genes in* Arabidopsis* has been found to improve tolerance to salt stress [54, 55]. This group of transcription factors was overrepresented in the roots of* B. napus* at an early stage after salt stress, indicating its possible role in regulating other important salt-responsive genes in this species.

In both the leaves and the roots, WRKY was the most abundant differentially regulated transcription factor in response to salt stress (Figure 7). Most of these upregulated DEGs showed high homology to previously identified WRKY genes in* Arabidopsis* and* B. napus*, for example,* BnWRKY3, BnWRKY4, BnWRKY11, BnWRKY29, BnWRKY40,* and* BnWRKY46*. Recently, many studies have shown that WRKY genes in wheat [56], soybean [57],* Tamarix hispida* [58], and* Arabidopsis* [59] are quickly induced at an early time point of salt stress. Salt-responsive WRKY genes identified in wheat, soybean, and* T. hispida* also enhance salinity tolerance when overexpressed in plants [56–58]. A total of 13* BnWRKY *genes, including* BnWRKY11* and* BnWRKY40*, in* B. napus* have been found to be responsive to both fungal pathogens and hormone treatments [60]. So far, there has been no finding of WRKY genes in* B. napus* responsive to salt stress and conferring salinity tolerance. The salt-responsive WRKY genes identified in this study are good candidates for further investigations for their potential roles in salinity tolerance in* B. napus*.

In addition, a large number of all-unigenes showing homology to various NAC transcription factor genes, for example,* BnNAC5-1*,* ANAC001*,* ANAC036*,* ANAC055,* and* ANAC090*, were found to be upregulated in the roots of* B. napus* at 1 h or 12 h after stress. It has been reported that six NAC genes (*BnNAC1-1*,* BnNAC5-1*,* BnNAC5-7*,* BnNAC5-8*,* BnNAC5-11,* and* BnNAC14*) were upregulated by various biotic and abiotic stresses such as mechanical wounding, insect feeding, fungal infection, cold shock, and dehydration [61]. Overexpression of* BnNAC5* in a* vni* T-DNA insertion mutant with salt-hypersensitive defect recovered the normal phenotype and many stress-responsive genes were enhanced in the* BnNAC5* overexpressing lines [46]. Upregulation of several NAC transcription factors in* B. napus* in the roots and the leaves is considered to be crucial for subsequent induction of stress responsive genes for tolerance.

### 3.9. Regulation of Ion Transporter Genes Involved in Ion Homeostasis

**Figure 8:**
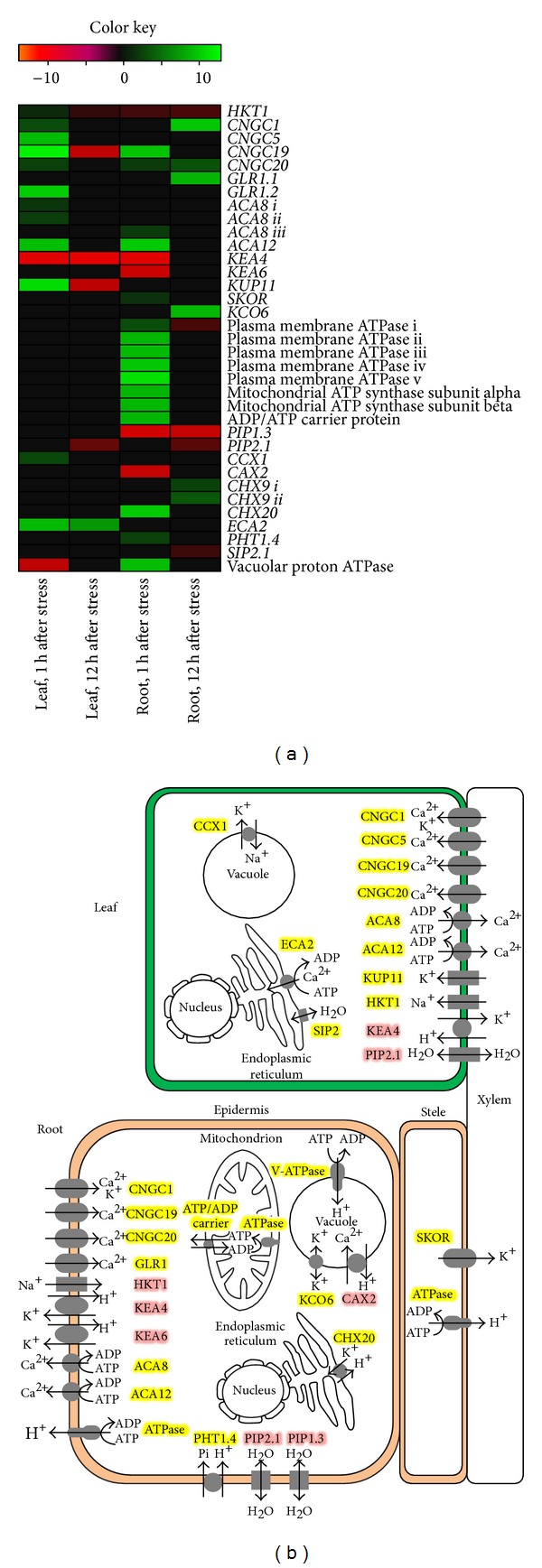
Regulation of transporter genes during salt stress. (a) Heat map depicting log2 fold change of differential expression of transporter genes in* B. napus*. (b) Cellular localization and functions of the regulated transporter in response to salt stress. Genes highlighted in yellow indicate upregulation while those highlighted in red indicate downregulation.

Among the 2,563 transporter genes annotated, 436 were either upregulated or downregulated by salt stress, that is, 231 transporter DEGs in the leaves, 261 DEGs in the roots, and 56 DEGs in both organs. Here, we focus on the regulation of ion transporters with potential functions in ion homeostasis in response to salt stress. The expression changes, subcellular localization, and functions are illustrated in Figure 8.

As shown in Figure 8,* HKT1* was found to be downregulated in the roots at an early stage after salt stress but upregulated in the leaves at 1 h after stress. According to our qRT-PCR result, the upregulation of* HKT1* was the most prominent in the leaves at 24 h (Figure 4). Na^+^ enters plant cells through HKT1, the high-affinity K^+^ transporter, and some other nonselective cation channels. Downregulation of* HKT1* in the roots can reduce toxic Na^+^ influx into the cytosol. However, upregulation of* HKT1* expression in the leaves would further enhance salt tolerance of this* B. napus* line. It has been demonstrated that* AtHKT1;1* is localized at the plasma membrane of xylem parenchyma cells in the shoots [62]. Previous study has shown both reduced phloem Na^+^ and elevated xylem Na^+^ in the shoots of* hkt1;1* mutants, thus indicating that AtHKT1;1 functions primarily to transport Na^+^ from the xylem into xylem parenchyma cells, at least in the shoots. Retrieval of Na^+^ from the xylem in the shoots reduces net Na^+^ influx into the shoots [63]. Based on this model, upregulation of* HKT1* in the leaves of this* B. napus* line possibly reduces Na^+^ transport into the leaves for salt tolerance.

Several transporters involved in cellular Ca^2+^ regulation exhibited differential expression. Expressions of many cyclic nucleotide-gated ion channels (CNGC) were found to be upregulated in both the leaves and the roots of* B. napus* (Figure 8). The glutamate receptors (GLR) were also upregulated in both organs by salt stress. Many of these CNGC and GLR molecules function in transporting Ca^2+^ into the cytosol. Some of these ion channels are also responsible for maintenance of cellular K^+^ content. Well-known initial responses of plant cells to salt stress are the generation of transient cytosolic Ca^2+^ flux and the subsequent activation of Ca^2+^ sensor proteins [62]. High concentrations of Na^+^ in external solution cause decreases in cellular K^+^ and Ca^2+^ contents of many plant species [64, 65], sometimes to certain extent resulting in K^+^ and Ca^2+^ deficiencies [66, 67]. It has been reported that the expression of* AtCNGC1* restored the Ca^2+^ conducting activity of a Ca^2+^ uptake-deficient mutant in response to mating pheromone [68]. The expression of* AtCNGC1* in K^+^ uptake deficient mutants of yeast and* Escherichia coli* enhanced growth of these mutants and increased intracellular [K^+^]. The increased expression of CNGC and GLR at an early stage of salt stress may demonstrate their putative contribution to cellular [K^+^] and [Ca^2+^] maintenance in* B. napus*.

Furthermore, two calcium-transporting ATPases homologous to* Arabidopsis ACA8* and* ACA12* were upregulated in the leaves and the roots in* B. napus *in response to salt treatment (Figure 8). The Ca^2+^-ATPase genes from* Arabidopsis* (*ACA12*) and the moss* Physcomitrella patens *(*PCA1*) have been shown to be upregulated after salt treatment [69, 70]. It has been proposed that Ca^2+^-ATPase is required to restore the [Ca^2+^]_cyt_ to prestimulus levels for generation of a specific transient increase in [Ca^2+^]_cyt_ essential for activation of signaling pathways related to abiotic stress [70]. It has been revealed that the moss* PCA1* loss-of-function mutants failed to generate a salt-induced transient Ca^2+^ peak and exhibited sustained elevated [Ca^2+^]_cyt_ in response to salt treatment, while WT moss showed transient Ca^2+^ elevation followed by restoration to prestress level [70]. The* PCA1* mutants were also more susceptible to salt stress and displayed a decreased expression level of stress-responsive genes [70]. Besides, a recent study has reported that the* aca8* and* aca10* mutant plants displayed decreased Flg22-triggered Ca^2+^ influx and ROS accumulation [71]. Therefore, an increased* ACA8* and/or* ACA12* expression in* B. napus* suggested their putative involvements in transient Ca^2+^ influx for subsequent activation of the signaling pathway essential for salinity tolerance.

K^+^ efflux antiporters (KEAs) were found to be downregulated in the leaves (*KEA4*) and in the roots (*KEA4*,* KEA6*) (Figure 8). The role of KEAs in ion homeostasis is poorly understood. Downregulation of these antiporters suggests restriction of the efflux of K^+^ out of the cellular compartment to maintain the cellular K^+^ level if it is localized at the plasma membrane. Localization of KEAs in* B. napus* or* Arabidopsis* and functional characterization using heterologous expression systems are necessary to determine their physiological roles. Another K^+^ transporter,* KUP11*, was upregulated at 1 h after stress in the leaves.* KUP11* has previously been found to be upregulated by salt stress in* Arabidopsis* shoots [72]. An increase of transcripts of* KUP1* and* KUP4* homologues has also been found in the ice plant (*Mesembryanthemum crystallinum*) during K^+^ starvation and salt exposure [73]. Upregulation of* KUP11* in the leaves in* B. napus* may contribute to maintenance of cytoplasmic K^+^ levels and turgor regulation during stress conditions, where external Na^+^ inhibits K^+^ uptake and cellular Na^+^ replaces K^+^. In addition, a shaker-type potassium channel (SKOR) was upregulated in the roots (Figure 8). In* Arabidopsis*, substantial salt-induced upregulation of* SKOR* in roots has been observed [72]. The SKOR channel mediates K^+^ release into the xylem channel [74]. Upregulation of both* SKOR* in roots and* AKT2/3* in shoots would also result in increased rates of K^+^ circulation through vascular tissue, pointing towards a long distance redistribution of K^+^ between the roots and shoots [75]. Upregulation of* SKOR* in the roots of* B. napus* is considered to be important in K^+^ homeostasis under saline conditions by promoting K^+^ circulation throughout the vascular tissue. In the roots of* B. napus*, a gene for vacuolar membrane-localized KCO6/TPK3 was also upregulated at an early stage of salt stress. In tobacco, a KCO6 homolog,* NtTPK1* was increased ~2-fold by salt stress [76]. Expression of* NtTPK1* in mutant* E. coli* deficient in three major K^+^ uptake systems rescued its phenotype [76]. Based on the above finding, upregulation of* KCO6/TPK3* in* B. napus* may be involved in transporting K^+^ into the cytosol resulting in alleviation of salt stress.

Expressions of a number of plasma membrane ATPase genes were upregulated in the roots of* B. napus* at 1 h after stress (Figure 8). In plant cells, primary active transport mediated by H^+^-ATPases and secondary transport mediated by channels and cotransporters are crucial to maintain characteristically high concentrations of K^+^ and low concentrations of Na^+^ in cytosol. Plasma-membrane H^+^-ATPase generates driving force for Na^+^ transport by SOS1 during salt stress [77]. Disruption of the root-endodermis-specific plasma-membrane H^+^-ATPase, that is,* AHA4*, in mutant* Arabidopsis* plants has also been found to enhance salt sensitivity [78]. The transcript levels of some H^+^-ATPases have also been shown to increase in response to salt stress [79].

In the roots, the transcription of mitochondrial ATP synthase *α*- and *β*-subunit together with ATP/ADP carrier was significantly upregulated at 1 h after stress (Figure 8). In wild-type yeast, NaCl stress increases both the mitochondrial F_1_F_0_-ATPase activity and expression of the F_1_F_0_-ATPase *α*-subunit [80]. Mitochondrial F_1_F_0_-ATPase activity in an aluminium tolerant wheat variety was also found to increase along with Al concentration although the *α*-subunit transcript remained constant [81]. Upregulation of both ATP synthase subunit and ATP/ADP carrier might correlate with the increase of F_1_F_0_-ATPase activity in the roots. Perhaps these upregulations facilitate increased ATP synthesis and the transport rate of ATP into cytosol for regulation of various cellular processes and active transport for adjustment to salt stress.

Salt treatment also downregulated the expression of plasma membrane-localized aquaporin PIP genes in both the leaves and the roots of* B. napus* at early stage of salt stress (Figure 8). Aquaporins are water channel proteins, which facilitate passive movement of water molecules down a water potential gradient [82]. Most of the water transport in plants occurs via aquaporins. Overexpression of* Arabidopsis* plasma membrane aquaporin,* PIP1b*, in tobacco enhances growth rate, transpiration rate, stomatal density, and photosynthetic efficiency under favorable growth conditions [83]. Conversely, overexpression of this aquaporin protein does not seem to have a beneficial effect under salt stress, and transgenic plants wilt faster than wild-type plants under drought stress [83]. Since overexpression of* PIP* results in enhanced symplastic water transport and increases stomatal density, such conditions are detrimental for plants growing under abiotic stresses. Downregulation of* PIP* in both the leaves and the roots might assist plants to cope with salt stress by limiting symplastic water transport and transpiration rate to prevent water loss.

A gene with high similarity to cation calcium exchanger,* CCX1*, in* Arabidopsis* was found to be upregulated in the leaves of this* B. napus* line. There are five* CCX* homologs (*CCX1* to* CCX5*) in* Arabidopsis* and both* CCX3* and* CCX4* have been functionally characterized [84]. Expression of* Arabidopsis AtCCX3* and* AtCCX4* in mutant yeast suppressed its mutant defective phenotypes in Na^+^, K^+^, and Mn^2+^ transport [84]. Subcellular localization indicates that AtCCX3 is accumulated in plant tonoplast [84]. Expression of* AtCCX3* increases in plants treated with NaCl, KCl, and MnCl_2_. Similar to* AtNHX1*-overexpressing plants,* AtCCX3*-expressing lines accumulate higher Na^+^ [84]. However, since* AtCCX3*-expressing plants did not appear to be salt tolerant,* AtCCX3*-expressing lines did not completely resemble* AtNHX1*-expressing plants. Therefore, it was hypothesized that overexpression of* AtCCX3* disrupts tonoplast V-type H^+^ translocating ATPase activity, causing a general disruption in pH homeostasis [84].* AtCCX3* has been suggested to be an endomembrane-localized H^+^ dependent K^+^ transporter with apparent Na^+^ and Mn^2+^ transport properties [84]. There is a possibility that* CCX1* expressed in* B. napus* functions in sequestration of Na^+^ into vacuoles and in ion homeostasis. Function characterization of* CCX1* in either* Arabidopsis* or* B. napus* is necessary to elucidate its involvement in salinity tolerance.

## 4. Conclusions

A comprehensive transcriptome of* B. napus* was characterized in both the leaves and roots by the Illumina sequencing technology. This transcriptome modulated by sudden increased salinity or salt shock expands our vision of the regulatory network involved in salinity adaptation in this amphidiploid. The candidate salt-responsive genes identified in* B. napus* included both the previously reported salt-responsive genes and some novel differentially regulated genes by salt stress, such as S1Fa-like transcription factor genes, some transporter genes, and some unknown protein genes, which will be a new resource for molecular breeding in crops. The molecular functions of many newly identified salt-responsive DEGs were still unknown. Transgenic assay, complementation assay, and subcellular localization could be employed to elucidate their possible contribution in salinity tolerance.

## Supplementary Material

Supplementary file 1 is primer pair for qRT-PCR analysis. Supplementary file 2 indicates expression fold change of salt responsive gene markers in B. napus. Supplementary file 3 and 4 are tables indicating summary of sequencing output and statistics for the unigene of B. napus assembled by SOAPdenovo, respectively. Supplementary file 5 reveals annotations of all-unigenes. Supplementary file 6 is a graph depicting length distribution of the all-unigenes with SwissProt or NR annotations. Supplementary file 7 and 8 are GO and KEGG ontology classifications of all-unigenes, respectively. Supplementary file 9 shows comparison of transcription factor families between B. napus, Arabidopsis and B. rapa. Supplementary file 10 indicates log2 fold-changes of DEGs in the leaves and the roots at 1h and 12h. Supplementary file 11 shows Venn diagrams depicting number of DEGs regulated in the leaves and the roots of B. napus. Supplementary file 12 shows the result of validation of DEGs by semi-qRT-PCR. Supplementary file 13 shows the comparison of expression fold-change for DEGs between N119 and Kirariboshi. Supplementary file 14 lists all over-represented GO terms in DEGs. Supplementary file 15, 16 and 17 indicate comparison of over-represented GO terms of “Biological Process”, “Molecular Function”, and “Cellular Component”, respectively in the leaves and the roots at 1h and 12h after stress. Supplementary file 18 lists all over-represented KEGG ontology in DEGs.

## Figures and Tables

**Table 1:** Statistics of the assembled unigene of leaf transcriptomes by Trinity method.

Length of unigenes (bp)	*BnLc*, 1 h	*BnLs*, 1 h	*BnLc*, 12 h	*BnLs*, 12 h
200–500	50,045	47,745	45,156	46,571
500–1000	26,937	26,584	26,585	27,125
1000–1500	10,689	11,320	12,355	12,286
1500–2000	4,016	4,721	5,645	5,397
>2000	2,278	2,987	3,984	3,494

Total unigenes	93,965	93,357	93,725	94,873
Mean length (bp)	646	682	732	711
N50 (bp)	861	933	1,025	986
Total length (bp)	60,699,848	63,664,784	68,579,723	67,428,136

**Table 2:** Statistics of the assembled unigene of root transcriptomes by Trinity method.

Length of unigenes (bp)	*BnRc*, 1 h	*BnRs*, 1 h	*BnRc*, 12 h	*BnRs*, 12 h
200–500	54,493	55,680	50,825	52,290
500–1000	28,077	27,208	27,408	27,384
1000–1500	12,275	12,638	13,450	12,097
1500–2000	5,606	5,928	6,539	5,137
>2000	3,525	4,573	4,872	3,392

Total unigenes	103,976	106,027	103,094	100,300
Mean length (bp)	682	703	737	679
N50 (bp)	956	1,014	1,072	947
Total length (bp)	70,938,630	74,504,289	76,016,194	68,138,136

**Table 3:** Statistics of the clustered all-unigene by CD-HIT-EST.

Length of unigenes (bp)	All-unigene
200–500	89,041
500–1000	37,426
1000–1500	18,623
1500–2000	9,230
>2000	7,217

Total unigenes	161,537
Mean length (bp)	693
N50 (bp)	1,039
Total length (bp)	111,953,629
