# Supplementary figures and images for: Comparative Transcriptome Analysis of Leaves and Roots in Response to Sudden Increase in Salinity in Brassica napus by RNA-seq
Source: Biomed Res Int. 2014 Aug 7;2014:467395. doi: 10.1155/2014/467395 (PMC4142189; doi:10.1155/2014/467395)

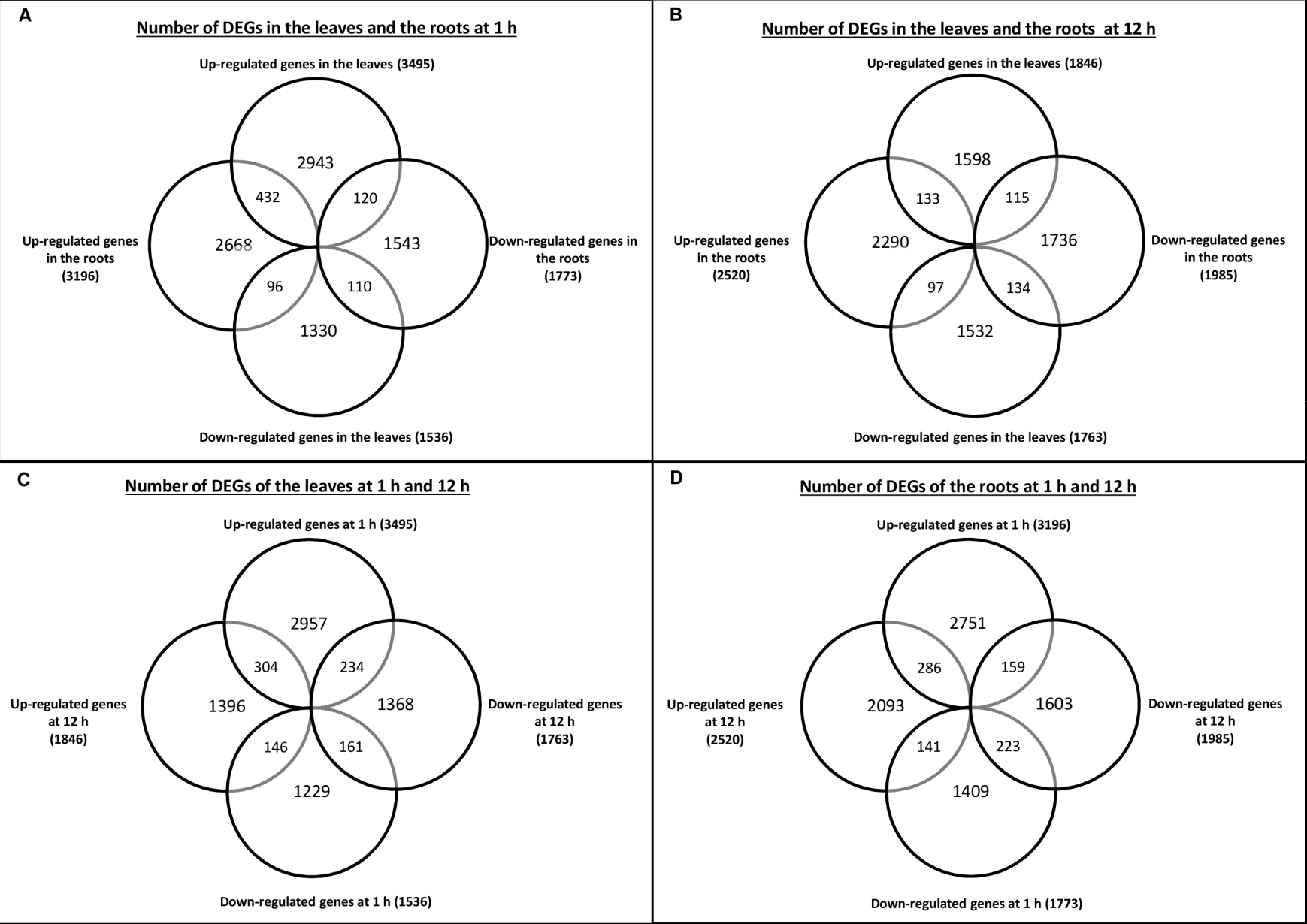

Supplement: Supplementary file 1 — is primer pair for qRT-PCR analysis. Supplementary file 2 indicates expression fold change of salt responsive gene markers in B. napus. Supplementary file 3 and 4 are tables indicating summary of sequencing output and statistics for the unigene of B. napus assembled by SOAPdenovo, respectively. Supplementary file 5 reveals annotations of all-unigenes. Supplementary file 6 is a graph depicting length distribution of the all-unigenes with SwissProt or NR annotations. Supplementary file 7 and 8 are GO and KEGG ontology classifications of all-unigenes, respectively. Supplementary file 9 shows comparison of transcription factor families between B. napus, Arabidopsis and B. rapa. Supplementary file 10 indicates log2 fold-changes of DEGs in the leaves and the roots at 1h and 12h. Supplementary file 11 shows Venn diagrams depicting number of DEGs regulated in the leaves and the roots of B. napus. Supplementary file 12 shows the result of validation of DEGs by semi-qRT-PCR. Supplementary file 13 shows the comparison of expression fold-change for DEGs between N119 and Kirariboshi. Supplementary file 14 lists all over-represented GO terms in DEGs. Supplementary file 15, 16 and 17 indicate comparison of over-represented GO terms of “Biological Process”, “Molecular Function”, and “Cellular Component”, respectively in the leaves and the roots at 1h and 12h after stress. Supplementary file 18 lists all over-represented KEGG ontology in DEGs. [file 467395.f1.zip › 467395.f1/Additional file 11.pdf]

# Additional file 12: Validation of DEGs by semi-qRT-PCR.

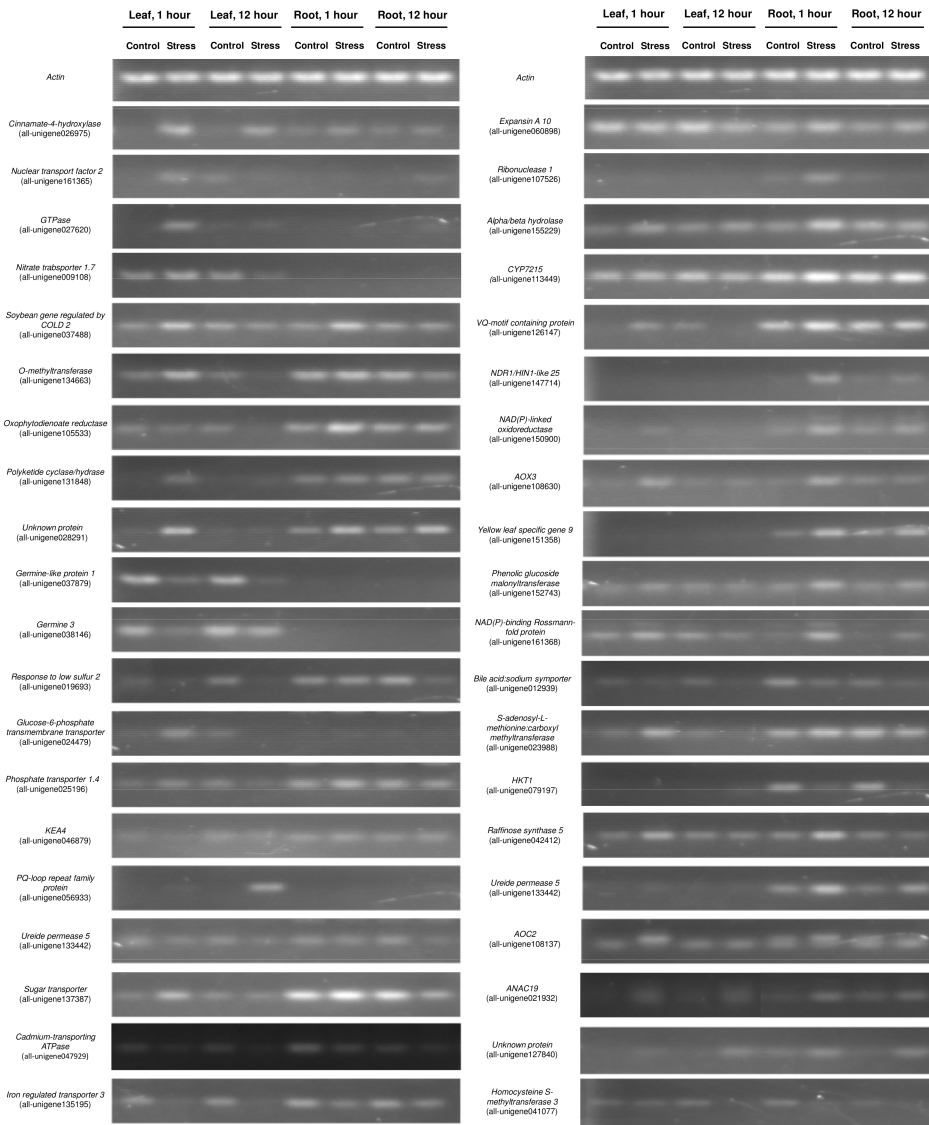

Supplement: Supplementary file 1 — is primer pair for qRT-PCR analysis. Supplementary file 2 indicates expression fold change of salt responsive gene markers in B. napus. Supplementary file 3 and 4 are tables indicating summary of sequencing output and statistics for the unigene of B. napus assembled by SOAPdenovo, respectively. Supplementary file 5 reveals annotations of all-unigenes. Supplementary file 6 is a graph depicting length distribution of the all-unigenes with SwissProt or NR annotations. Supplementary file 7 and 8 are GO and KEGG ontology classifications of all-unigenes, respectively. Supplementary file 9 shows comparison of transcription factor families between B. napus, Arabidopsis and B. rapa. Supplementary file 10 indicates log2 fold-changes of DEGs in the leaves and the roots at 1h and 12h. Supplementary file 11 shows Venn diagrams depicting number of DEGs regulated in the leaves and the roots of B. napus. Supplementary file 12 shows the result of validation of DEGs by semi-qRT-PCR. Supplementary file 13 shows the comparison of expression fold-change for DEGs between N119 and Kirariboshi. Supplementary file 14 lists all over-represented GO terms in DEGs. Supplementary file 15, 16 and 17 indicate comparison of over-represented GO terms of “Biological Process”, “Molecular Function”, and “Cellular Component”, respectively in the leaves and the roots at 1h and 12h after stress. Supplementary file 18 lists all over-represented KEGG ontology in DEGs. [file 467395.f1.zip › 467395.f1/Additional file 12.pdf]

Additional file 12: Comparison of expression fold-change for DEGs between N119 and Kirariboshi.

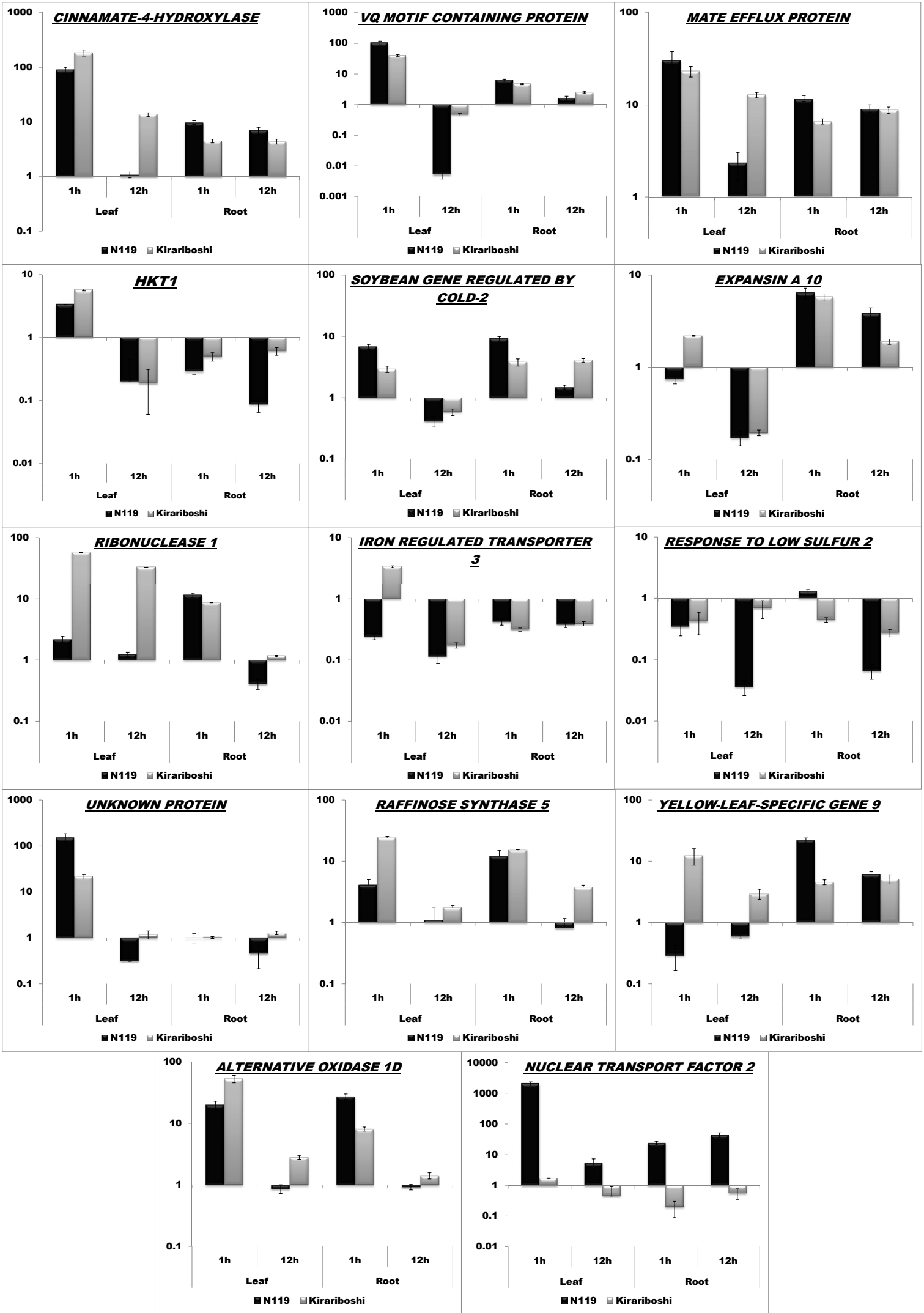

Supplement: Supplementary file 1 — is primer pair for qRT-PCR analysis. Supplementary file 2 indicates expression fold change of salt responsive gene markers in B. napus. Supplementary file 3 and 4 are tables indicating summary of sequencing output and statistics for the unigene of B. napus assembled by SOAPdenovo, respectively. Supplementary file 5 reveals annotations of all-unigenes. Supplementary file 6 is a graph depicting length distribution of the all-unigenes with SwissProt or NR annotations. Supplementary file 7 and 8 are GO and KEGG ontology classifications of all-unigenes, respectively. Supplementary file 9 shows comparison of transcription factor families between B. napus, Arabidopsis and B. rapa. Supplementary file 10 indicates log2 fold-changes of DEGs in the leaves and the roots at 1h and 12h. Supplementary file 11 shows Venn diagrams depicting number of DEGs regulated in the leaves and the roots of B. napus. Supplementary file 12 shows the result of validation of DEGs by semi-qRT-PCR. Supplementary file 13 shows the comparison of expression fold-change for DEGs between N119 and Kirariboshi. Supplementary file 14 lists all over-represented GO terms in DEGs. Supplementary file 15, 16 and 17 indicate comparison of over-represented GO terms of “Biological Process”, “Molecular Function”, and “Cellular Component”, respectively in the leaves and the roots at 1h and 12h after stress. Supplementary file 18 lists all over-represented KEGG ontology in DEGs. [file 467395.f1.zip › 467395.f1/Additional file 13.pdf]

Additional file 2: Expression fold change of salt responsive gene markers in *B. napus*.

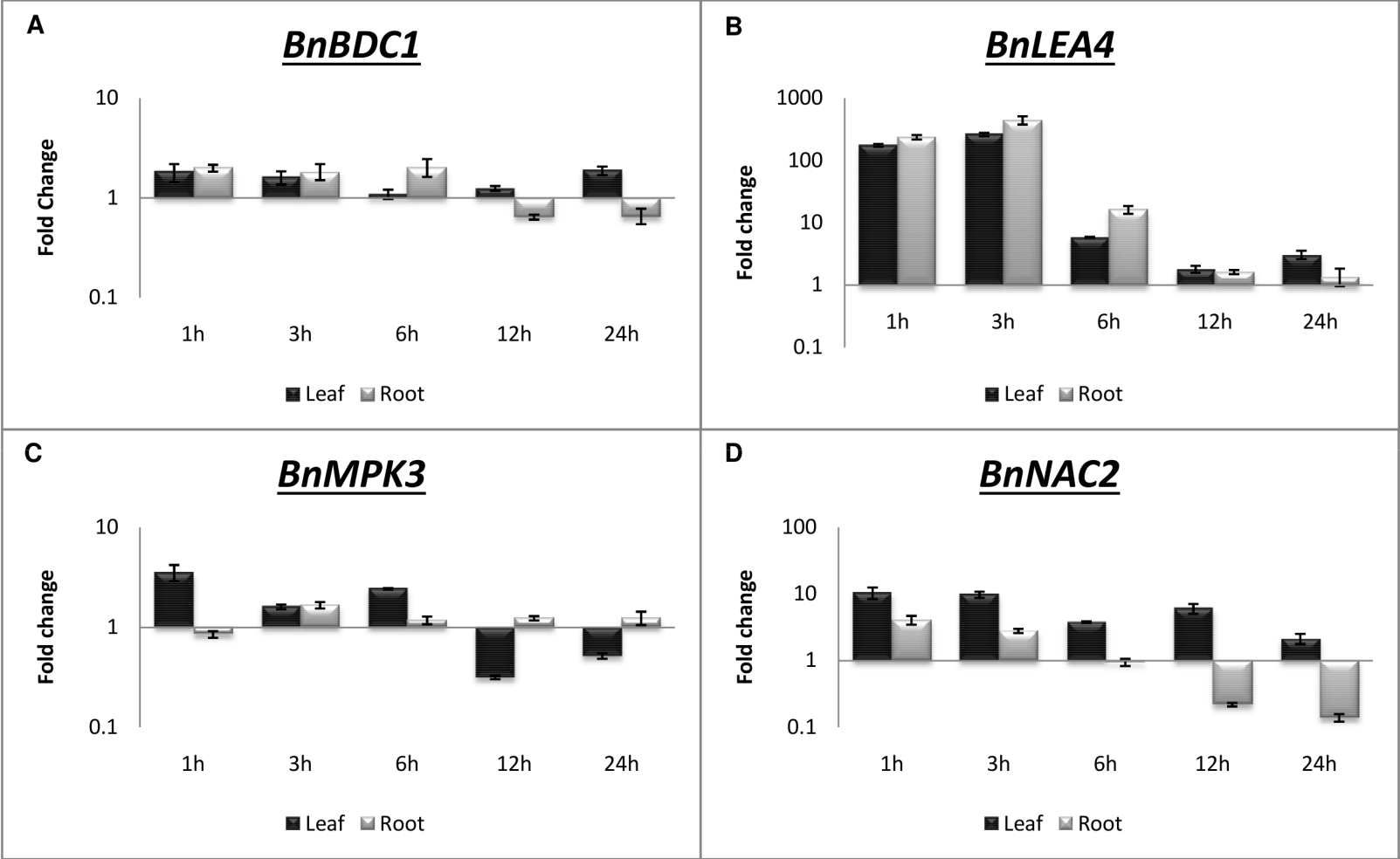

Supplement: Supplementary file 1 — is primer pair for qRT-PCR analysis. Supplementary file 2 indicates expression fold change of salt responsive gene markers in B. napus. Supplementary file 3 and 4 are tables indicating summary of sequencing output and statistics for the unigene of B. napus assembled by SOAPdenovo, respectively. Supplementary file 5 reveals annotations of all-unigenes. Supplementary file 6 is a graph depicting length distribution of the all-unigenes with SwissProt or NR annotations. Supplementary file 7 and 8 are GO and KEGG ontology classifications of all-unigenes, respectively. Supplementary file 9 shows comparison of transcription factor families between B. napus, Arabidopsis and B. rapa. Supplementary file 10 indicates log2 fold-changes of DEGs in the leaves and the roots at 1h and 12h. Supplementary file 11 shows Venn diagrams depicting number of DEGs regulated in the leaves and the roots of B. napus. Supplementary file 12 shows the result of validation of DEGs by semi-qRT-PCR. Supplementary file 13 shows the comparison of expression fold-change for DEGs between N119 and Kirariboshi. Supplementary file 14 lists all over-represented GO terms in DEGs. Supplementary file 15, 16 and 17 indicate comparison of over-represented GO terms of “Biological Process”, “Molecular Function”, and “Cellular Component”, respectively in the leaves and the roots at 1h and 12h after stress. Supplementary file 18 lists all over-represented KEGG ontology in DEGs. [file 467395.f1.zip › 467395.f1/Additional file 2.pdf]

**Additional file 6: Length distribution of the all-unigenes with SwissProt or NR annotations.**

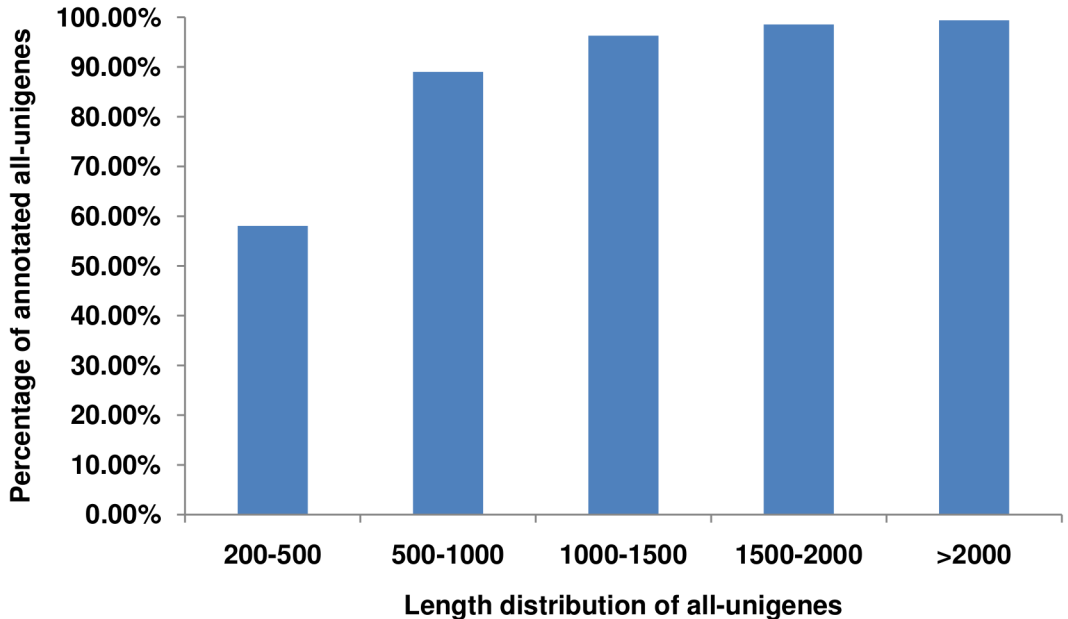

Supplement: Supplementary file 1 — is primer pair for qRT-PCR analysis. Supplementary file 2 indicates expression fold change of salt responsive gene markers in B. napus. Supplementary file 3 and 4 are tables indicating summary of sequencing output and statistics for the unigene of B. napus assembled by SOAPdenovo, respectively. Supplementary file 5 reveals annotations of all-unigenes. Supplementary file 6 is a graph depicting length distribution of the all-unigenes with SwissProt or NR annotations. Supplementary file 7 and 8 are GO and KEGG ontology classifications of all-unigenes, respectively. Supplementary file 9 shows comparison of transcription factor families between B. napus, Arabidopsis and B. rapa. Supplementary file 10 indicates log2 fold-changes of DEGs in the leaves and the roots at 1h and 12h. Supplementary file 11 shows Venn diagrams depicting number of DEGs regulated in the leaves and the roots of B. napus. Supplementary file 12 shows the result of validation of DEGs by semi-qRT-PCR. Supplementary file 13 shows the comparison of expression fold-change for DEGs between N119 and Kirariboshi. Supplementary file 14 lists all over-represented GO terms in DEGs. Supplementary file 15, 16 and 17 indicate comparison of over-represented GO terms of “Biological Process”, “Molecular Function”, and “Cellular Component”, respectively in the leaves and the roots at 1h and 12h after stress. Supplementary file 18 lists all over-represented KEGG ontology in DEGs. [file 467395.f1.zip › 467395.f1/Additional file 6.pdf]

Additional file 7: GO classification of *B. napus* transcriptome.

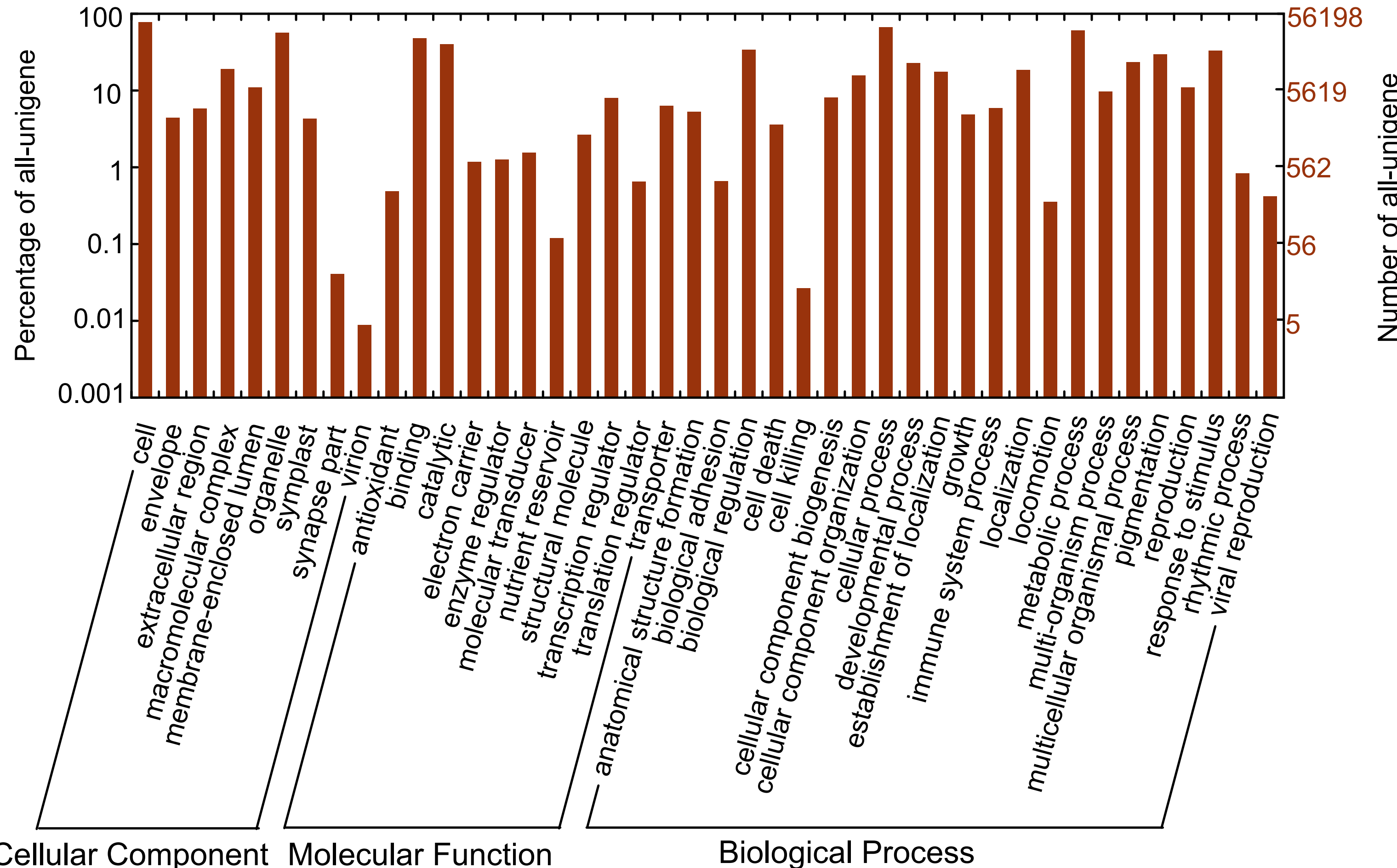

Supplement: Supplementary file 1 — is primer pair for qRT-PCR analysis. Supplementary file 2 indicates expression fold change of salt responsive gene markers in B. napus. Supplementary file 3 and 4 are tables indicating summary of sequencing output and statistics for the unigene of B. napus assembled by SOAPdenovo, respectively. Supplementary file 5 reveals annotations of all-unigenes. Supplementary file 6 is a graph depicting length distribution of the all-unigenes with SwissProt or NR annotations. Supplementary file 7 and 8 are GO and KEGG ontology classifications of all-unigenes, respectively. Supplementary file 9 shows comparison of transcription factor families between B. napus, Arabidopsis and B. rapa. Supplementary file 10 indicates log2 fold-changes of DEGs in the leaves and the roots at 1h and 12h. Supplementary file 11 shows Venn diagrams depicting number of DEGs regulated in the leaves and the roots of B. napus. Supplementary file 12 shows the result of validation of DEGs by semi-qRT-PCR. Supplementary file 13 shows the comparison of expression fold-change for DEGs between N119 and Kirariboshi. Supplementary file 14 lists all over-represented GO terms in DEGs. Supplementary file 15, 16 and 17 indicate comparison of over-represented GO terms of “Biological Process”, “Molecular Function”, and “Cellular Component”, respectively in the leaves and the roots at 1h and 12h after stress. Supplementary file 18 lists all over-represented KEGG ontology in DEGs. [file 467395.f1.zip › 467395.f1/Additional file 7.pdf]

**Additional file 8: KEGG ontology classification of *B. napus* transcriptome.**

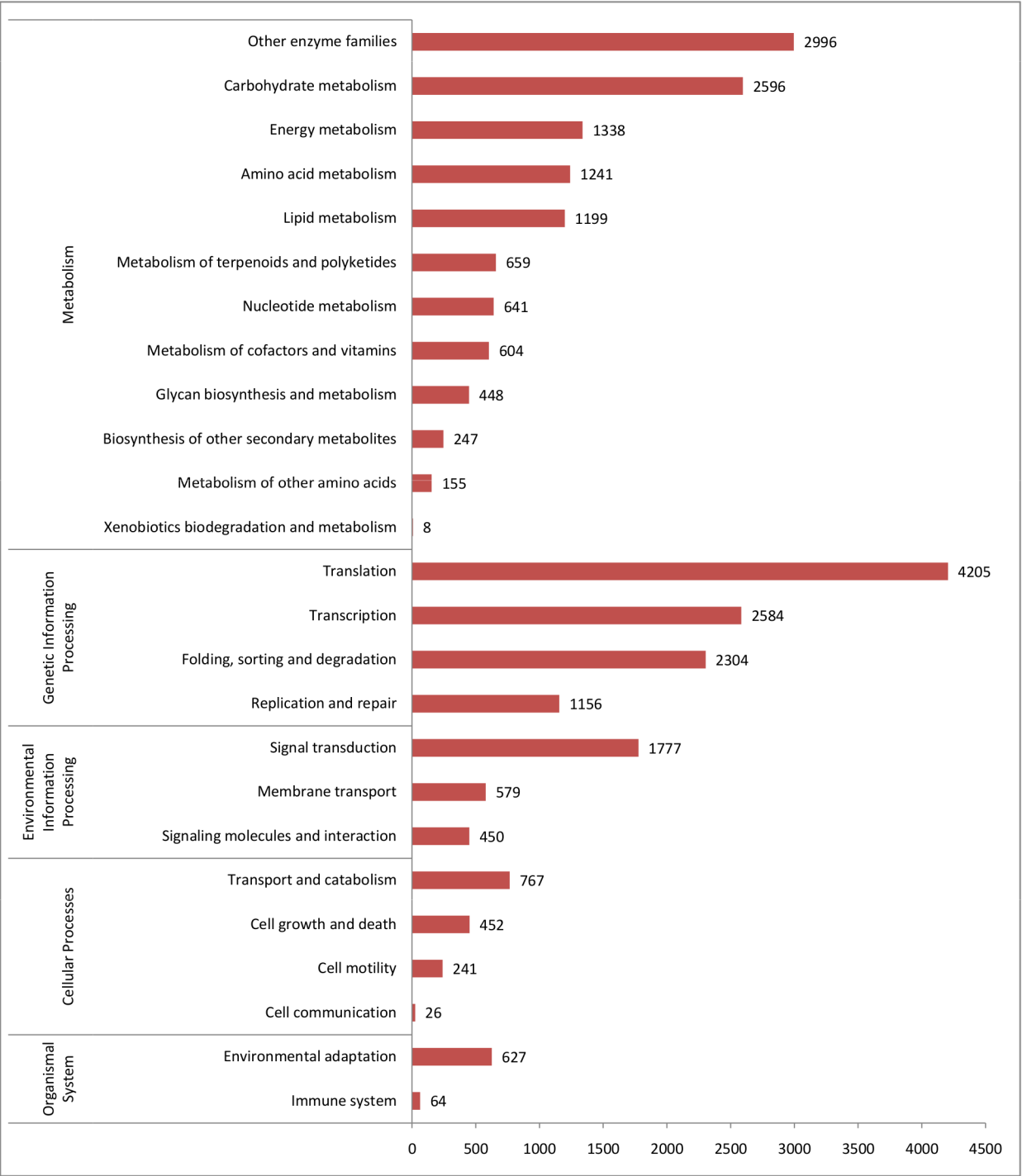

Supplement: Supplementary file 1 — is primer pair for qRT-PCR analysis. Supplementary file 2 indicates expression fold change of salt responsive gene markers in B. napus. Supplementary file 3 and 4 are tables indicating summary of sequencing output and statistics for the unigene of B. napus assembled by SOAPdenovo, respectively. Supplementary file 5 reveals annotations of all-unigenes. Supplementary file 6 is a graph depicting length distribution of the all-unigenes with SwissProt or NR annotations. Supplementary file 7 and 8 are GO and KEGG ontology classifications of all-unigenes, respectively. Supplementary file 9 shows comparison of transcription factor families between B. napus, Arabidopsis and B. rapa. Supplementary file 10 indicates log2 fold-changes of DEGs in the leaves and the roots at 1h and 12h. Supplementary file 11 shows Venn diagrams depicting number of DEGs regulated in the leaves and the roots of B. napus. Supplementary file 12 shows the result of validation of DEGs by semi-qRT-PCR. Supplementary file 13 shows the comparison of expression fold-change for DEGs between N119 and Kirariboshi. Supplementary file 14 lists all over-represented GO terms in DEGs. Supplementary file 15, 16 and 17 indicate comparison of over-represented GO terms of “Biological Process”, “Molecular Function”, and “Cellular Component”, respectively in the leaves and the roots at 1h and 12h after stress. Supplementary file 18 lists all over-represented KEGG ontology in DEGs. [file 467395.f1.zip › 467395.f1/Additional file 8.pdf]
